# Infection by Different Clades of *Candidozyma auris* in a *Galleria mellonella* Model: Determining Virulence Levels

**DOI:** 10.1007/s11046-026-01082-5

**Published:** 2026-06-18

**Authors:** Gabriel Davi Marena, Alejandro Lopez, Gabriela Corrêa Carvalho, Javier Pemán, Jose Manuel Perez-Royo, Victor Garcia-Bustos, Ángel González, Paula Muñoz Brell, María Dolores Pérez Ruiz, Lara Zaragoza Macian, Carmen Vicente Saez, Antonia Avalos Mansilla, Tais Maria Bauab, Marlus Chorilli, Alba Ruiz-Gaitán

**Affiliations:** 1 Health Research Institute La Fe, Avenida Fernando Abril Martorell, 106 Torre A 6a Planta, 46026 Valencia, Spain; 2 Department of Medical Microbiology, University and Polytechnic La Fe Hospital, Valencia, Spain; 3 Department of Drugs and Medicines, School of Pharmaceutical Sciences, São Paulo State University (UNESP), Araraquara, São Paulo, 14800-903 Brazil; 4 School of Pharmaceutical Sciences, Department of Biological Sciences, São Paulo State University (UNESP), Araraquara, São Paulo, 14800-903 Brazil; 5 Department of Pathological Anatomy, La Fe Hospital, Valencia, Spain; 6 Basic and Applied Research Group (MICROBA), School of Microbiology, University of Antioquia, Medellín, Colombia; 7 Federal University of Mato Grosso Do Sul (UFMS), Faculty of Pharmaceutical Sciences, Food and Nutrition (FACFAN), Campo Grande, Brazil

**Keywords:** *Candida auris*, *Candidozyma auris*, *Galleria mellonella*, Infection, Virulence, Granulomas, Biofilms

## Abstract

**Introduction:**

*Candidozyma auris* (formerly *Candida auris*) is an emerging yeast that causes bloodstream infections, especially in immunocompromised patients, and presents high resistance and virulence rates. To date, six clades have been established worldwide and the number of outbreaks caused by this microorganism has been increasing every year, causing concern in the medical community. Therefore, this study investigated the heterogeneity among clades of *C. auris* by evaluating the virulence profile and mechanism of infection using an *in vivo* model of *Galleria mellonella.*

**Methods:**

G*. mellonella* was infected with different clades (I, II, III and IV) of *C. auris, C. albicans* ATCC 5341 and *C. parapsilosis* ATCC 22019 for virulence and histopathologic evaluation.

**Results:**

Aggregative strains of *C. auris* InP13 (I) and VEN C6 (IV) had a greater rate of melanization and larval mortality among the *C. auris* isolates, therefore, being the most aggressive strains. *C. albicans* caused the most melanization among all strains at the highest inoculum concentration (10^6^ cells/mL). Histopathologic examination showed a greater number of granulomas in the lower and upper extremities of *G. mellonella*. The granulomas ranged from 0.07–0.11 nm in diameter. All strains showed biofilms adhering to larval tissue, which was more evident for InP13, VEN C6 and *C. albicans*. Infiltration of tissues by yeasts, pseudohyphae and chlamydospores (a resistance structure formed by *C. albicans* in stress environments) morphotypes were observed.

**Conclusion:**

The aggregative strains were more virulent and had a greater ability to form biofilms and granulomas, showing heterogeneity among the different *C. auris* clades.

## Introduction

*Candidozyma auris* (formerly *Candida auris*) is an emerging, pathogenic, and multidrug-resistant yeast known to cause systemic infections and outbreaks, with high mortality rates in patients with comorbidities or those undergoing intensive care. However, *C. auris* infections have undergone drastic changes, since these outbreaks are increasing every day, and the number of patients who have lost their lives is worrying. Once installed in a healthcare facility, *C. auris* persists for a long time, is highly resistant to antifungal therapy and disinfectants, and rapidly spreads among susceptible patients [1, 2]. In 2025, the European Centre for Disease Prevention and Control (ECDC) published a report highlighting the worrying increase in *C. auris* cases in European hospitals, posing a serious threat to patients and the healthcare system. The report emphasizes the importance of early diagnosis and transmission control to prevent rapid and widespread spread [3].

Currently, epidemiology suggests six distinct clades of *C. auris*: Clade I isolated in South Asia, Clade II from in East Asia, Clade III in South Africa, and Clade IV isolated in South America. The clade V has already been reported in Iran and is characterized by approximately 200,000 nucleotides that differ from the other clades. According to this information, there is heterogeneity among *C. auris* species, which can be justified by geographic location and recurrent climate changes [4]. Finally, and more recently, clade VI was detected and sequenced in Singapore in 2023 [5].

The antifungal resistance and susceptibility profile has been observed to be variable. Furthermore, difficult identification by conventional and molecular methods, resistance to common disinfectants, unclear dissemination mechanisms, and uncertain environmental niches are considered factors that hinder the control of *C. auris* worldwide [6, 7]. Moreover, virulence mechanisms such as the ability to form biofilms, surface adhesion or phospholipases and proteinases production are of concern to researchers worldwide [8, 9].

An *in vivo* model using *Galleria mellonella* has become one of the most widely used and effective assays in antimicrobial and infection studies. With similar characteristics to mammalian cells and their immune response, the *G. mellonella* model aids in antimicrobial discovery by evaluating compounds and the mechanism of pathogens infection through virulence and immunological assays [10–13]. An additional feature of this insect is melanin production: the enzyme phenol oxidase oxidises catecholamines (e.g., L-DOPA) to quinones that polymerise into melanin, a dark pigment that contributes to immune defence. Melanin encapsulates and immobilises fungi, restricting gas exchange and nutrient access, and can ultimately kill the microorganism [14].

Therefore, this study evaluated the virulence potential and infection mechanism of four clinical isolates belonging to clades I, II, III, and IV of *C. auris* in a *G. mellonella* model.

## Material and Methods

### Fungal Strains

Four clinical strains were assayed: *C. auris* VPCI479/P13 (CLADE I – InP13), *C. auris* CBS10913 (CLADE II – JAP 1), *C. auris* CBS 15603 (CLADE III – SP96), *C. auris* VEN C6 (CLADE IV – VEN C6); in addition, two reference strains of *Candida* species were also included: *C. albicans* SC 5134 and *C. parapsilosis* ATCC 22019.

### Aggregation Assays

An aliquot of colony forming units of each strain was transferred into a 0.85% saline solution to observe the presence or absence of yeast clusters under an optical microscope.

### Virulence Assays

An *in vivo* model of *G. mellonella* (250–350 mm in height) was used for the virulence test. The larvae were first separated into groups of 20 and kept in a petri dish at 37 °C for 24 h for environmental adaptation. Before infection, a sterile toothpick containing 70% alcohol was used to clean the proleg region, the area where the yeast inoculum would be injected. Inoculum of each *Candida* spp. was standardised to a final concentration of 10^4^, 10^5^ and 10^6^ cells/larvae in a PBS solution containing 20 µg/mL ampicillin (Sigma Aldrich, Steinheim, North Rhine-Westphalia, Germany), as described by Mesa-Arango et al. [15]. Then, using a Hamilton Microliter™ syringe, 10 µL of each *Candida* ssp. inoculum was injected into the penultimate proleg of each larva and incubated at 37 °C (20/group). Every 24 h of incubation, for 10 days, dead larvae were quantified. The experiment was performed in triplicate and on different days.

### Evaluation of Melanization Process

**Fig. 1:**
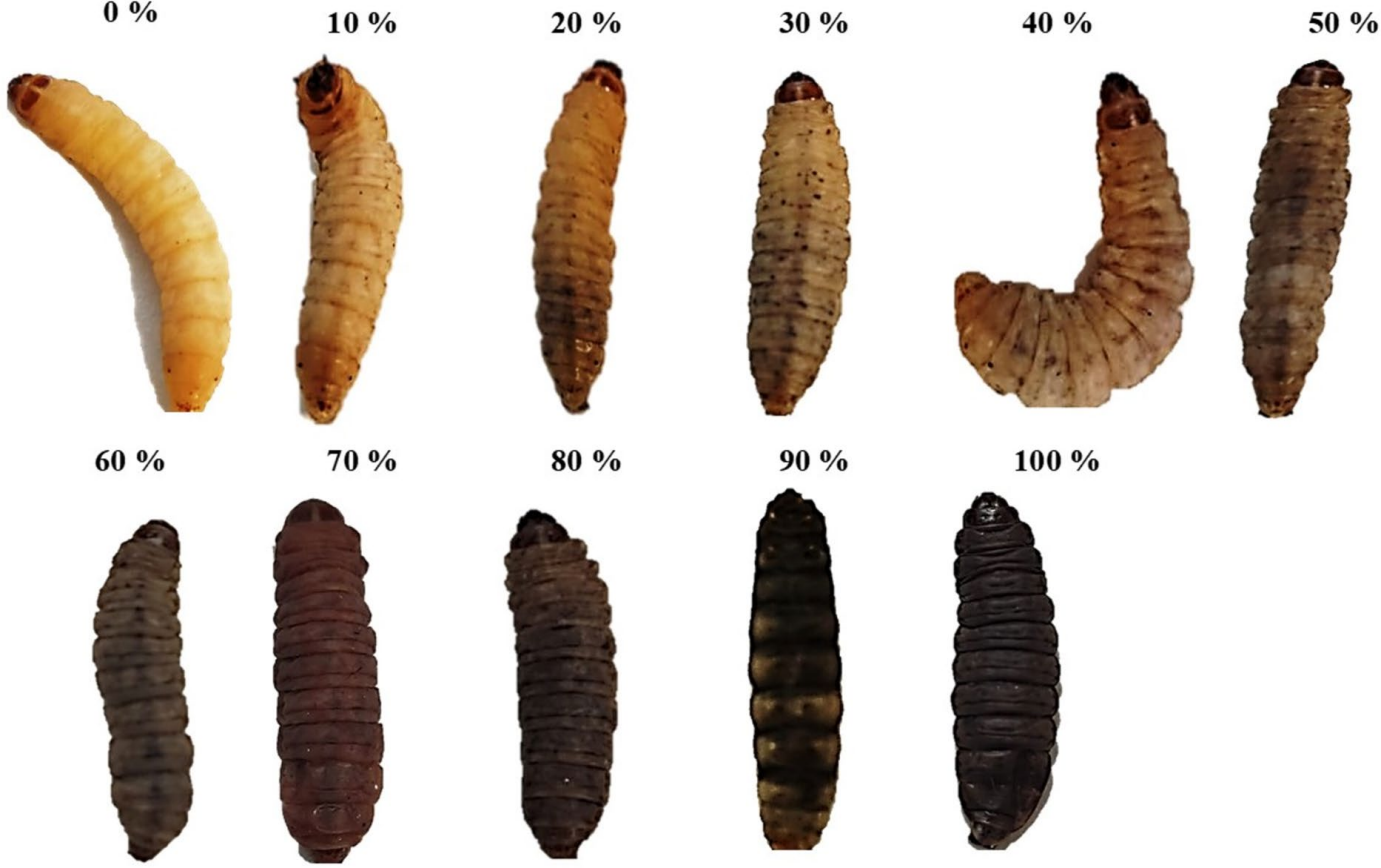
Melanin level scale in a model of *Galleria mellonella* infected with *Candida* spp

The melanization assay followed the infection model described above and was evaluated for 10 days. Each larva was observed individually and compared with the uninfected control group (cream colour, no changes). Melanization was quantified as a percentage based on larval colour, as shown in Fig. 1, and according to the protocol of Garcia-Bustos et al. [16]. The inoculum concentrations evaluated were 10^4^, 10^5^, and 10^6^ cells/larva. However, 10^4^ cells/larva did not cause melanization and is therefore not shown in the graph.

### Histopathological Analysis

To carry out the histopathology analysis, larvae were inoculated with 10^4^ cells/larvae of each *C. auris* isolate and with 10^5^ cells/larvae for non-*C. auris* strains and kept at 37ºC. Two larvae were collected 120 h after infection, euthanized in 5% ethanol, and placed into tubes containing 10 mL of 4% formalin for 20 days to preserve the tissue and fixation. thereafter, larvae were cut sagittal and fixed in paraffin. Hematoxylin–eosin (HE) staining was performed for the morphological analysis of organelles, granulomas, and cellular infiltration Periodic acid-Schiff staining (PAS) was performed to assess tissue morphology, granulomas, yeast morphology, and yeast infiltration into tissues. Uninfected larvae were processed similarly for comparative purposes. One infected larva of each yeast strain was selected for analysis in a Philips IMS Scanners to observe the regions with the highest number of granulomas. The mean diameter of the granulomas was measured using a Slide Viewer program.

### Ethical Statement

*Galleria mellonella* is not classified as an animal under current European and international regulations, and therefore its use does not require ethical approval by institutional animal care and use committees. This model provides a valuable, ethical, and high throughput platform for preliminary *in vivo* testing, helping to reduce the use of vertebrate animals in line with the 3Rs principles (Replacement, Reduction, and refinement) in research.

### Statistical Analysis

For virulence/survival analysis we used One-way analysis of variance (ANOVA) was used for comparison between infected and uninfected control groups using GraphPad Prism 8.0.

## Results

### Aggregative and Non-aggregative Phenotype Determination of *C. auris* Isolates

Before the biological tests, microscopic analysis was carried out to determine the aggregative and non-aggregative strains and, according to the analysis, it was possible to determine that the aggregative strains were clade I, III and IV (InP13, SP96 and VEN C6, respectively) with the JAP 1 isolate (Clade II) being the only non-aggregative one.

### *Candida auris* Clades I and IV Appear to be More Lethal for *G. mellonella* Larvae

**Fig. 2:**
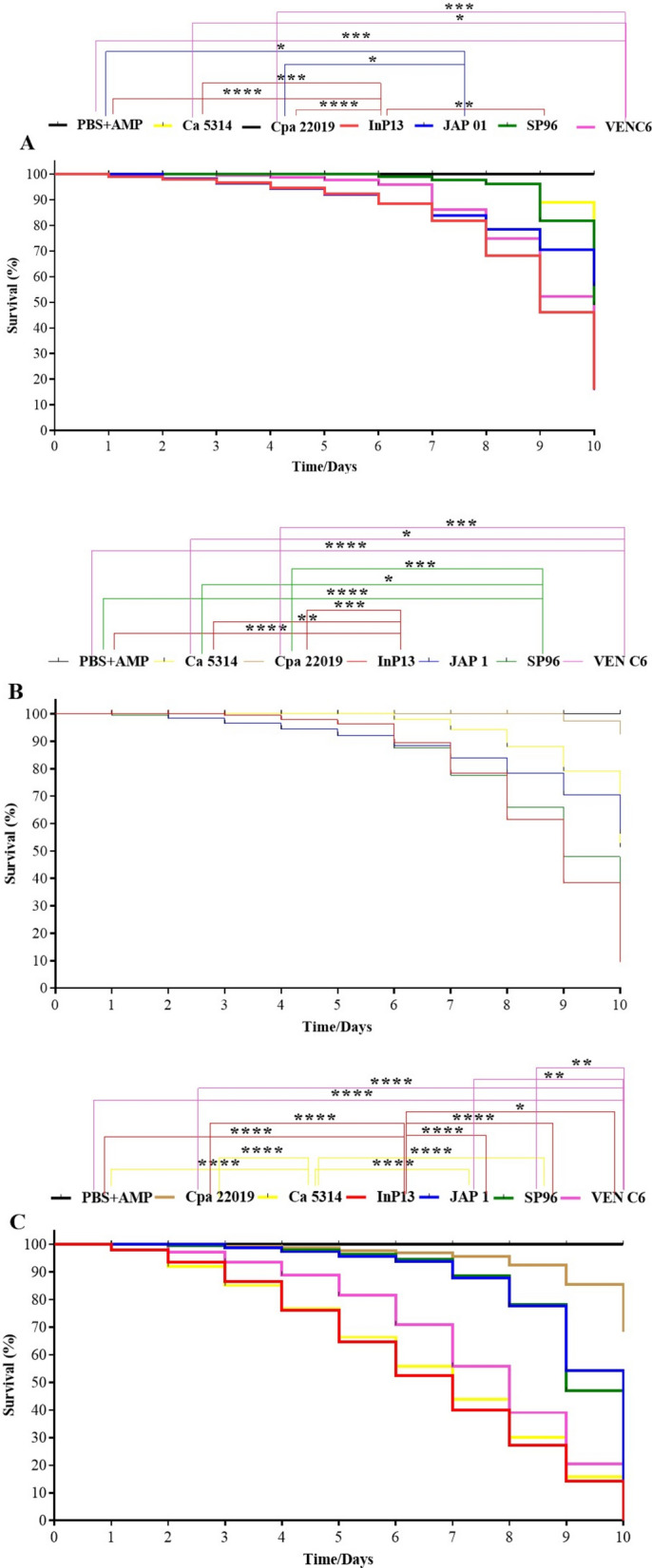
Survival analysis of *G. mellonella* larvae infected with *Candida* spp. **A** *G. mellonella* larvae inoculated with 10^4^; **B** 10^5^; and **C** 10^6^ yeasts/larva. InP13 (clade I): *C. auris* VPCI479/P13; JAP 1 (clade II): *C. auris* CBS10913; SP96 (clade III): *C. auris* CBS 15603; VEN C6 (clade IV): *C. auris* VEN C6; Ca 5314: *C. albicans* ATCC 5314; Cp 22019: *C. parapsilosis* ATCC 22019; PBS + AmP: Phosphate Buffered Saline + Ampicillin at 20 µg/mL. (*): *p* < *0.05*; (**): *p* < *0.005*; (***): *p* < *0.001*; (****): *p* < *0.0001*

As shown in Fig. 2, the aggregative isolates InP13, SP96 and VEN C6 were the ones that caused the highest mortality rate and were considered the most virulent (*p* < *0.0001*). Among the aggregative isolates, InP13 was the most virulent and its mortality rate was directly related to an increase in yeast concentration. The mortality rate in larvae infected with InP13 was greater than 50% after 7, 9, and 9 days postinfection when inoculum 10^6^, 10^5^ and 10^4^ yeasts/larva were used, respectively. After 10 days of infection, mortality was 83.9, 90.5 and 100% in groups infected with inoculum 10^4^, 10^5^ and 10^6^ yeasts/larvae, respectively (*p* < *0.0001*).

VEN C6 (clade IV) was the second most virulent strain among *C. auris* strains, causing mortality greater than 50% on days 8, 9, and 9 postinfection when inoculum of 10^6^, 10^5^, and 10^4^ yeasts/larva were used, respectively. After 10 days of infection, mortality was 84.3, 90.5, and 100% in groups infected with inoculum 10^4^, 10^5^, and 10^6^ yeasts/larvae, respectively. SP96 (clade III, aggregative) and JAP 1 (clade II, non-aggregative) were the least virulent strains, with JAP 1 having the lowest mortality. After 10 days of infection with SP96, mortality was 50.9, 85.6, and 95.3% in groups infected with inoculum 10^4^, 10^5^, and 10^6^ yeasts/larvae, respectively. For the group infected with JAP 1, the mortality rate after 10 days was 43.6, 43.3, and 89.2% with inoculum 10^4^, 10^5^, and 10^6^ yeasts/larvae, respectively.

Furthermore, *C. albicans* 5314 caused the highest number of larval deaths, with an inoculum of 10^6^ yeast/larva. After 10 days of infection, mortality was 28.8, 48.5, and 100% in groups infected with inoculums 10^4^, 10^5^, and 10^6^ yeast/larvae, respectively. *C. parapsilosis* causes lower mortality at 10^6^ yeasts/larva, with approximately 30% lethality after 10 days of infection.

### Melanization Process in *G. mellonella* did not Differ Among the Different Strains (Belonging to Clades I-IV) of *C. auris*

**Fig. 3:**
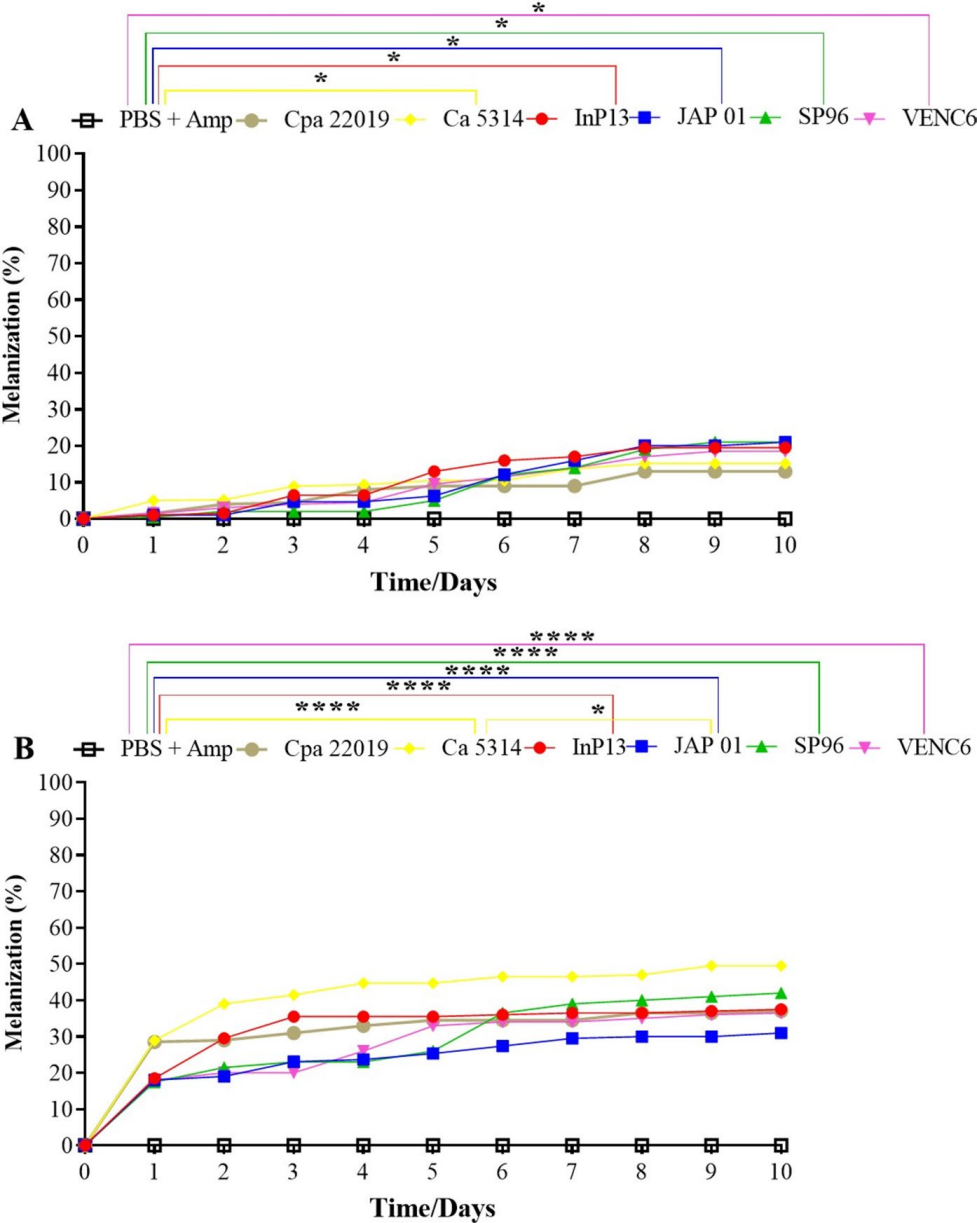
Melanin production in *G. mellonella* infected with *Candida* spp. **A**: 10^5^ yeasts/larvae; **B**: 10^6^ yeasts/larvae; InP13: *C. auris* VPCI479/P13; JAP 1: *C. auris* CBS10913; SP96: *C. auris* CBS 15603; VEN C6: *C. auris* VEN C6; Ca 5314: *C. albicans* ATCC 5314; Cp 22019: *C. parapsilosis* ATCC 22019; PBS + AmP: Phosphate Buffered Saline + Ampicillin at 20 µg/mL. (*): *p* < *0.05*; (****): *p* < *0.0001*

Figure 3 shows the results of the melanization assay and as observed, there was no evidence of melanin levels for the inoculum of 10^4^ yeasts/larva with any of the clinical isolates. However, the injection of 10^5^ yeasts/larva caused a total of 13% (*C. parapsilosis*) 15.2% (*C. albicans*), 18.5% (VEN C6), 19.5% (InP13), 21% (JAP 1) and 21% (SP96) of melanization in the larval body (Fig. 3A). However, there was no statistical difference between the infected groups, only compared to the group treated with PBS + AmP, with a *p* < *0.05* (Fig. 3A). Noteworthy, it was observed that the 10^6^ yeasts/larva inoculum, as shown in Fig. 3B, the group infected with *C. albicans* showed 49.5% of melanization at the end of the period evaluated, being the species that caused the most melanization process (*p* < *0.0001)*. On the other hand, JAP 1 was the strain with least melanization, with 31% (*p* = *0.0121*). Finally, larvae infected with the higher inoculum (10^6^ cells) of VEN C6, *C. parapsilosis*, InP13, and SP96 showed 36.5, 37, 37.5, and 42% of melanization, respectively, with a significant difference compared to the uninfected group (*p* < *0.0001*).

### Histological Analysis of *Galleria mellonella*

**Fig. 4:**
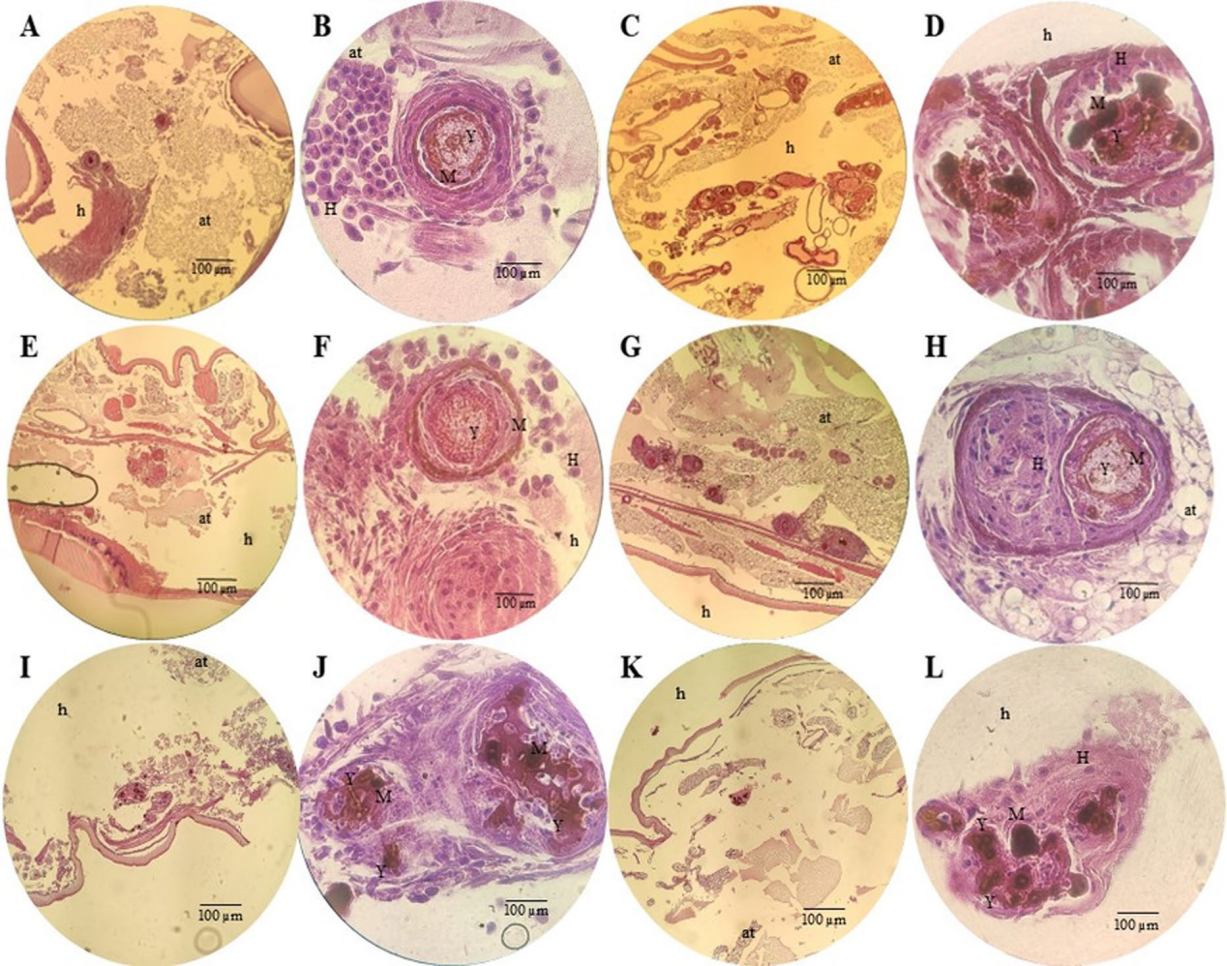
Histological analysis of *G. mellonella* infected with *C. auris* and non-*C. auris* and stained with HE. **A**: 10 × InP13; **B**: InP13 100x; **C**: JAP 1 10x; **D**: JAP 1 100x; **E**: SP96 10x; **F**: SP96 100x; **G**: VEN C6 10x; **H**: VEN C6 100x; **I**: *C. albicans* 5134 10x; **J**: *C. albicans* 5134 100x; **K**: *C. parapsilosis* 22019 10x; **L**: *C. parapsilosis* 22019 × 100.(h): hemolymph; (at): adipose tissue; (H): haemocytes; (Y): yeast; (M): melanin

**Fig. 5:**
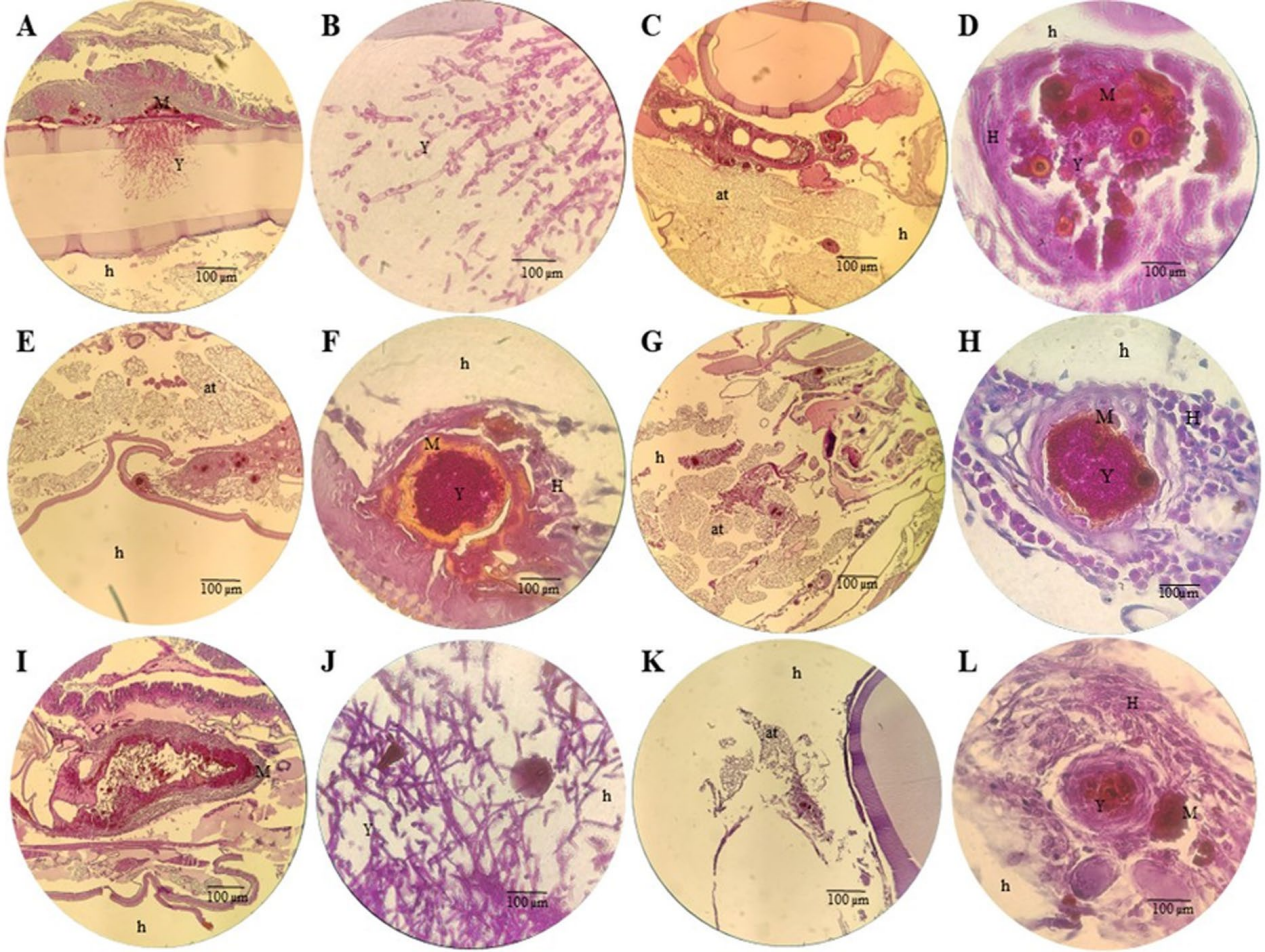
Histological analysis of *G. mellonella* infected with *C. auris* and non-*C. auris* and stained with PAS. **A**: 10 × InP13; **B**: InP13 100x; **C**: JAP 1 10x; **D**: JAP 1 100x; **E**: SP96 10x; **F**: SP96 100x; **G**: VEN C6 10x; **H**: VEN C6 100x; **I**: *C. albicans* 5134 × 10x; **J**: *C. albicans* 5134 100x; **K**: *C. parapsilosis* 22019 10x; **L**: *C. parapsilosis* 22019 100x. (h): hemolymph; (at): adipose tissue; (H): haemocytes; (Y): yeast; (M): melanin

Figures 4 and 5 show the histological analysis of *G. mellonella* infected with *Candida* spp. Tissue sections were stained with HE and PAS, respectively.

The images show the development of yeast clusters (Y) surrounded by haemocytes (H), forming granuloma structures throughout the larval tissue. During infectious and inflammatory processes, large amount of melanin is produced, creating orange/brown areas (M). Granulomas are found throughout the larva body and are more abundant in the lower extremities (tail) and the upper extremities (head).

Larvae infected with InP13 showed granulomas in their tissues with an intense immunological response; moreover, these structures were surrounded by adipose tissue in the area between the upper and lower intestines, known as the transitory region (Fig. 4A–B). InP13 isolate produced yeast infiltration in the organelles, which was caused by an intense inflammatory response and adipose tissue in the lower intestine (Fig. 5A–B). Furthermore, yeasts with defined morphologies stood out, with intense budding and hyphae/pseudohyphae formation were observed (Fig. 5B). JAP 1 isolate caused granulomas in different regions of the larval body, particularly in the head (Fig. 4C–D) and intermediate regions (Fig. 5C–D). A high level of melanization and tissue necrosis was observed (Fig. 4D), and it can also be noted that yeast-like structures were internalized by haemocytes and surrounded by adipose tissue (Fig. 5C). Granulomas induced by the SP96 isolate were found in the tail of *G. mellonella*, close to the cuticle (Figs. 4 and 5 E–F); moreover, granuloma showed haemocytes with clusters of yeast surrounded by melanin (Figs. 4F and 5F). Granuloma formed by VEN C6 isolate surrounded by haemocytes and adipose tissue were found in the upper intestine (Figs. 4 G-H) and tail (Figs. 5 G-H). Invasive infection with a high degree of inflammation, melanization, and tissue necrosis was observed by *C. albicans* (Figs. 4 and 5, I–J). A well-defined yeast structures were observed with PAS staining, which allowed to observe budding cells (Figs. 5J) or yeasts associated with melanin formation and hyphae/pseudohyphae formation process (Figs. 4J). A cluster of small melanized regions surrounded by haemocytes was observed between the upper and lower intestines in larvae infected with *C. parapsilosis* (Fig. 4K–L). Another highlight was that granulomas surrounded by adipose tissue and haemocytes were found dispersed in the haemolymph of the lower intestine (Fig. 5 K–L).

**Fig. 6:**
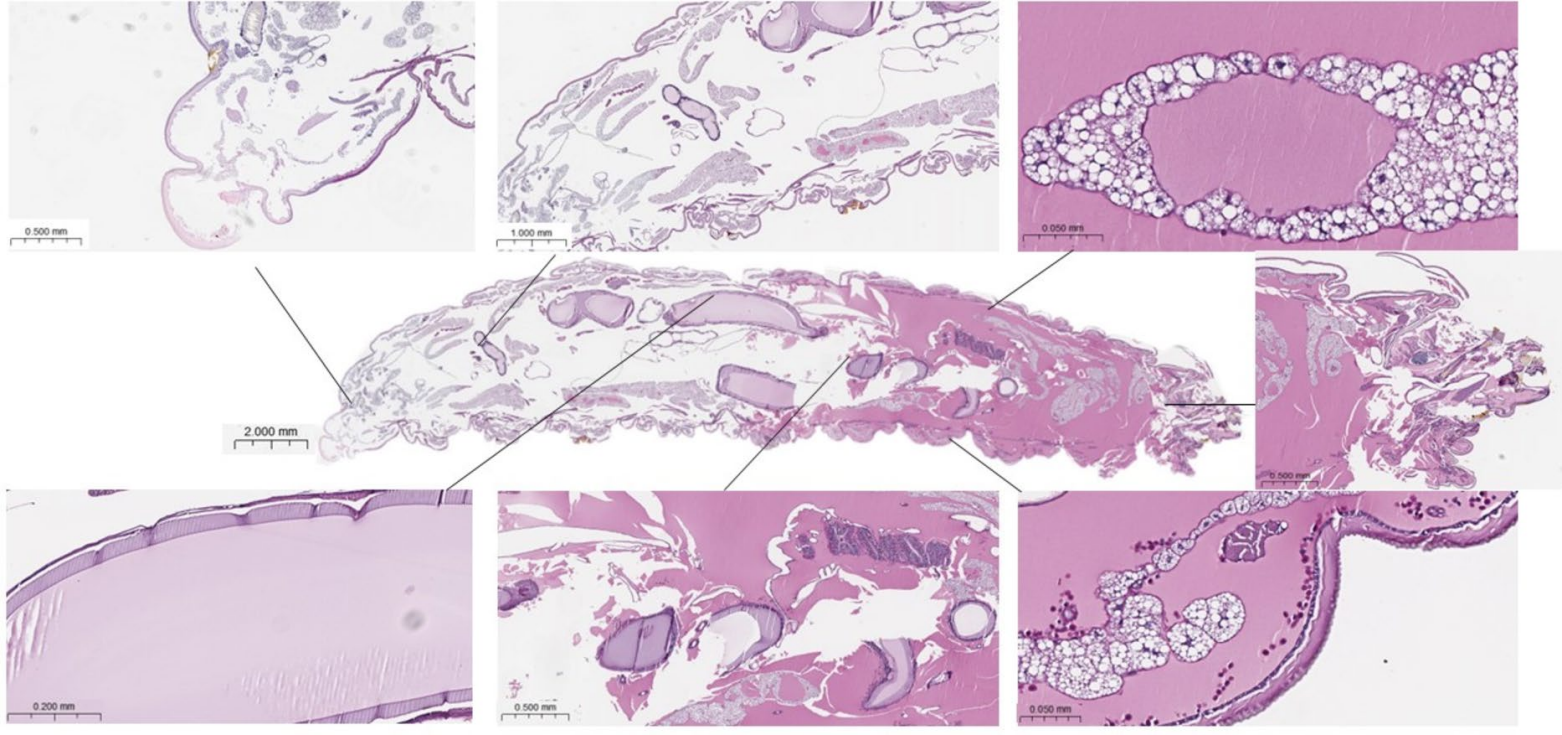
Histological anatomy of *G. mellonella* without infection and stained with PAS. **A**: Head; **B**: Chest; **C**: adipose tissue; **D**: Lower region–tail; **E**: Intestinal wall; **F**: Transitory region; **G**: Proleg of the cuticle

Figure 6 shows the anatomy of *G. mellonella* without infection. The head and thorax are in the upper extremity regions (A and B). The intermediate region contains most of the organs, such as the trachea, intestine, muscle tissue, adipose tissue, and most of the haemolymph (E). The junction between the intermediate and lower ends was in the transitional region (F). Finally, the tail (E) of the lower region consisted of adipose tissue (C) and other organelles responsible for excretion. The prolegs (G) are located on the posterior surface of the larva and are composed of spore fragments that are responsible for locomotion on the surface.

**Fig. 7:**
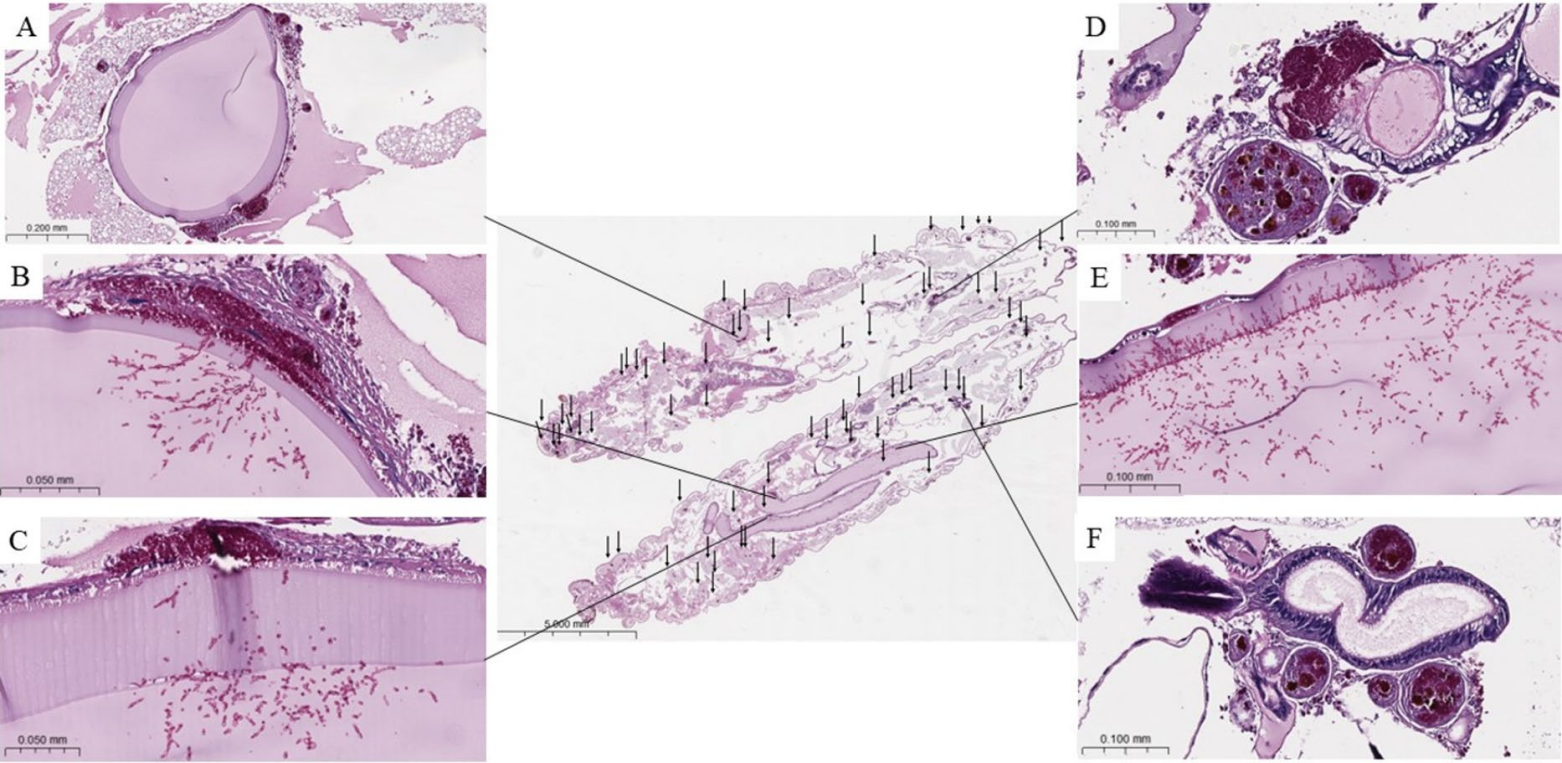
Distribution of yeast aggregates and granulomas in *G. mellonella* tissue infected with *C. auris* InP13. Images analyzed by SlideViewer software; **A**–**C**: 20x; **D** and **F**:10x; **E**: 30x; Black arrows indicate granulomas or yeast aggregates; PAS staining

**Fig. 8:**
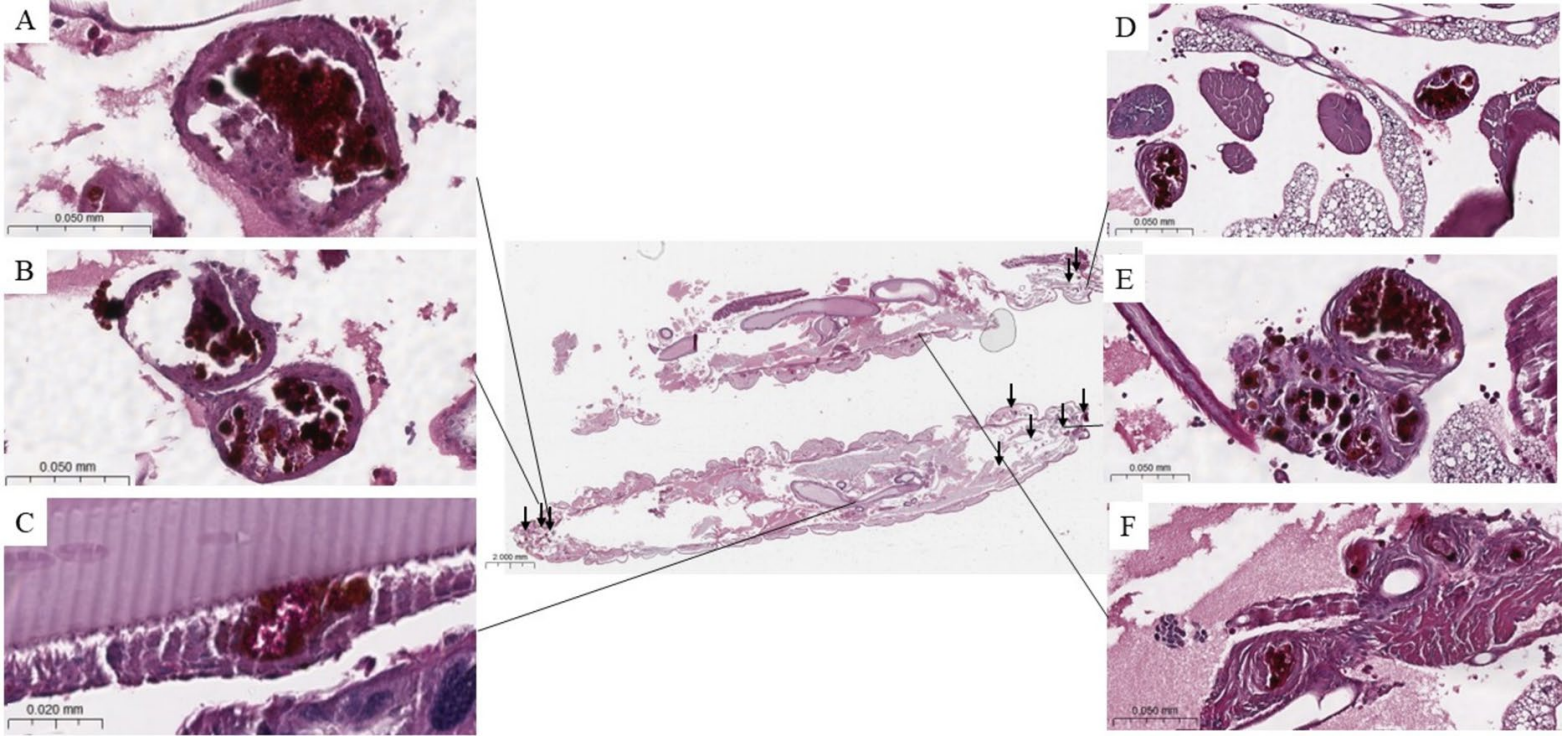
Distribution of yeast aggregates and granulomas in *G. mellonella* tissue infected with *C. auris* JAP 1. Images analyzed by SlideViewer software; **A**–**C**: 20x; **D**:5x; **E**–**F**: 10x; Black arrows indicate granulomas or yeast aggregates; PAS staining

**Fig. 9:**
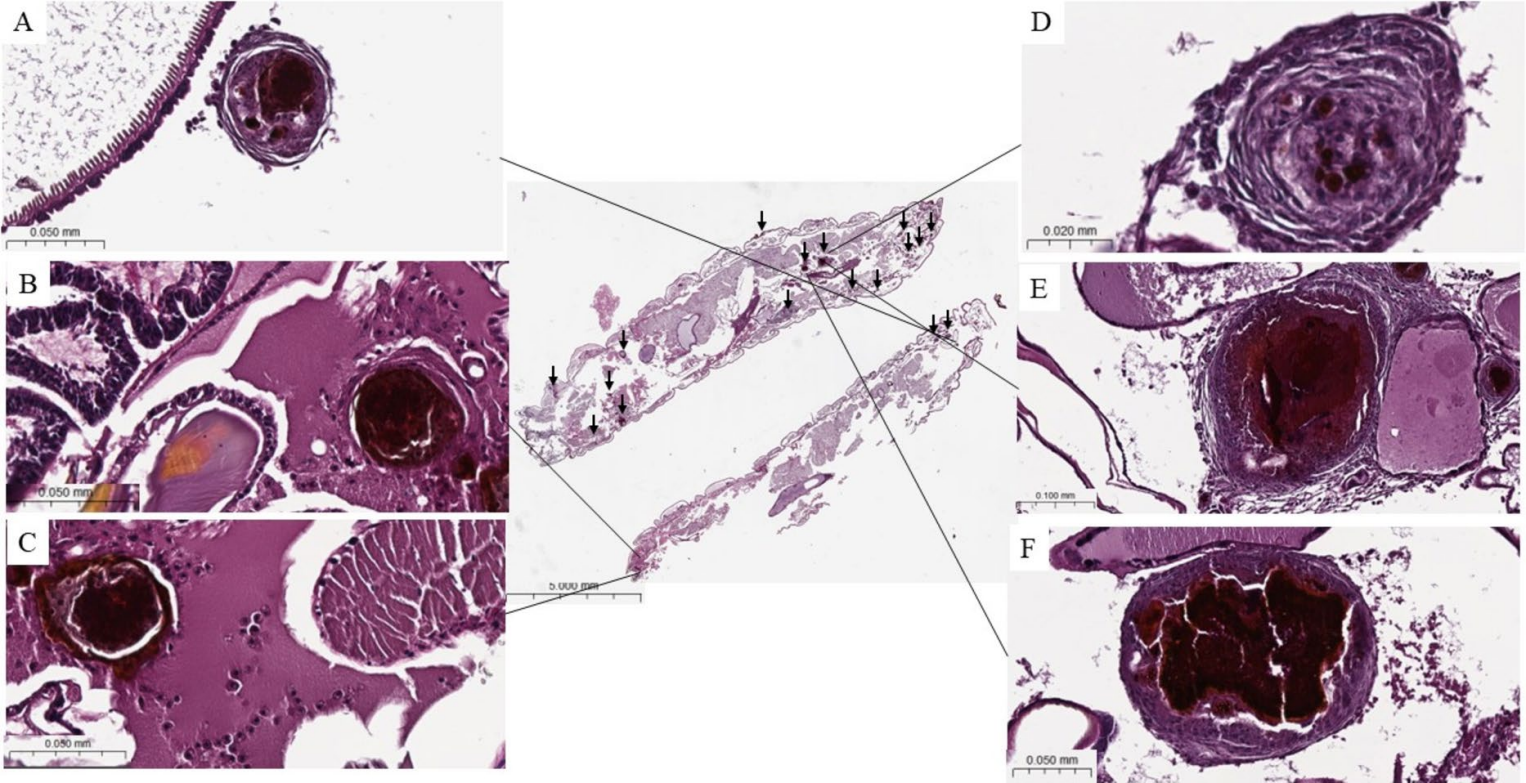
Distribution of yeast aggregates and granulomas in *G. mellonella* tissue infected with *C. auris* SP96. Images analyzed by SlideViewer software; **A**–**C** and **E**–**F**: 20x; **D**:10x; Black arrows indicate granulomas or yeast aggregates; PAS staining

**Fig. 10:**
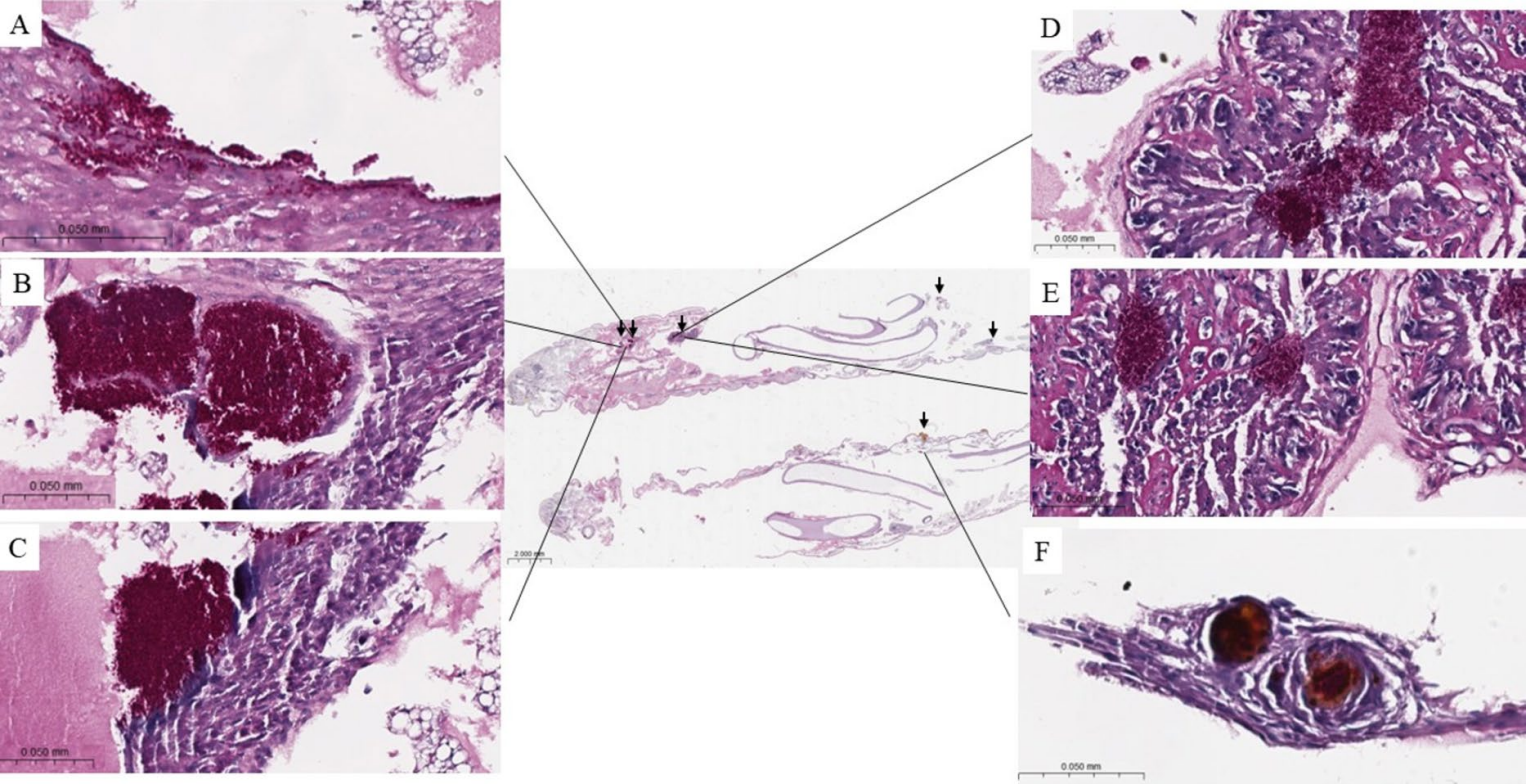
Distribution of yeast aggregates and granulomas in *G. mellonella* tissue infected with *C. auris* VEN C6. Images analyzed by SlideViewer software; **A**: 10x; **B**–**F**:20x; Black arrows indicate granulomas or yeast aggregates; PAS staining

**Fig. 11:**
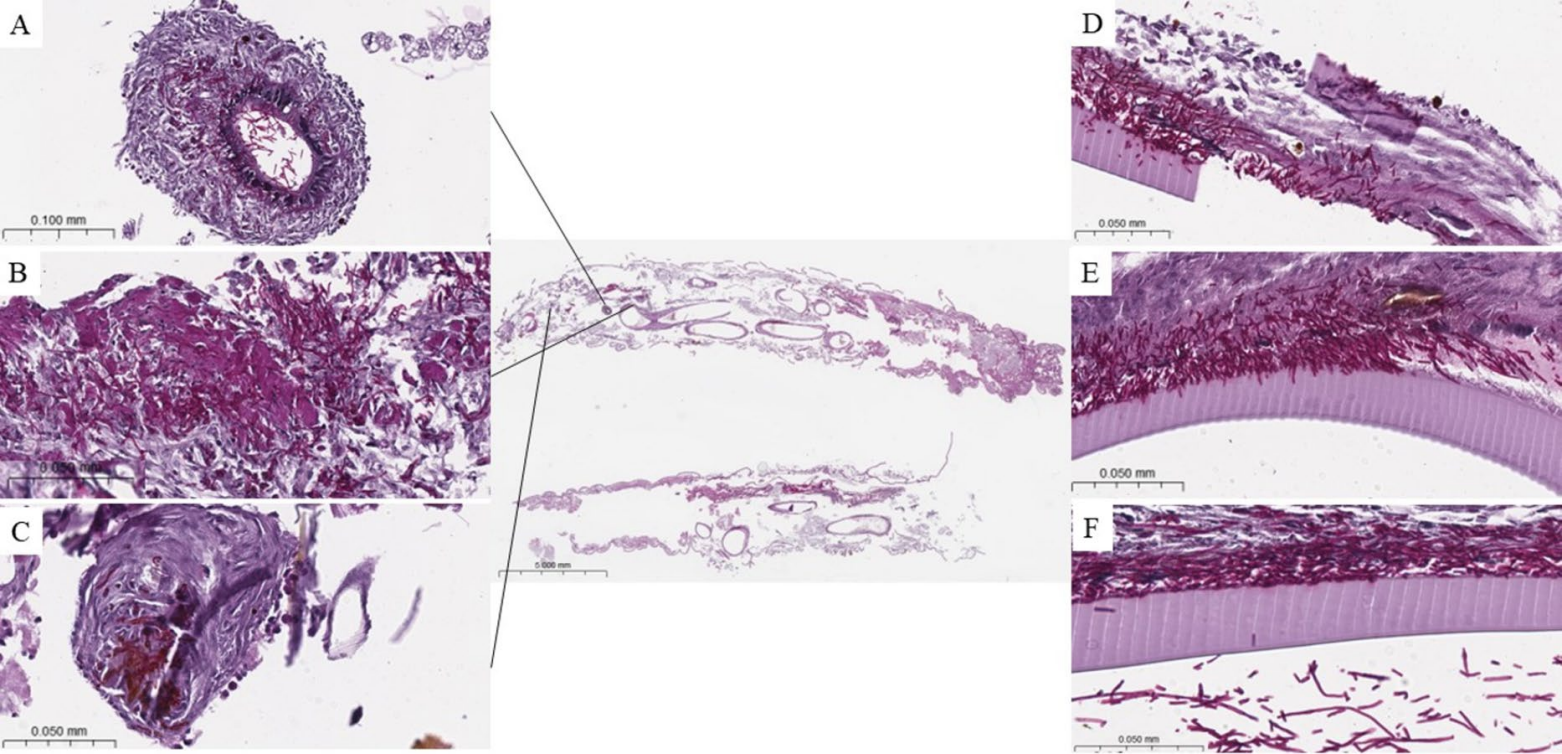
Distribution of yeast aggregates and granulomas in *G. mellonella* tissue infected with *C. albicans.* Images analyzed by SlideViewer software; **A** and **E**: 20x; **B**–**C** and **E**–**F**: 30x; **D**: 10x; Black arrows indicate granulomas or yeast aggregates; PAS staining

Figures 7–11 show granulomas, yeast aggregates, and infiltration located in different regions of *G. mellonella*. As shown in Fig. 7, several granulomas were observed in the larvae’s tissue infected with the InP13 isolate. In addition, yeast infiltration in the intestinal wall located in the transitional region (the region between the lower and upper extremities) was observed. This infiltration was followed by the presence of yeast aggregates with budding formation (Fig. 7 B, C, and E). Yeast aggregates forming a uniform biofilm-like community attached to the intestinal wall and surrounded by haemocytes throughout the outer cavity in the transitional region (Fig. 7 A) and lower end (Figs. 7 D and F) were observed. Granulomas with diameters of 0.083 ± 0.047 mm were observed throughout the tissue.

JAP1 infection induced formation of biofilm-like yeast aggregates located between the outer intestinal wall and surrounded by haemocytes in the transition region (Fig. 8 C). Granulomas caused by JAP 1 were found in the lower and upper extremities, formed between adipose tissue (Fig. 8 F), close to the cuticle lower proleg (Figs. 8 D and E) and head (Fig. 8 A and B), with diameters of approximately 0.11 ± 0.036 mm.

Granulomas formed by SP96 infection were observed between the lower and upper regions, reaching a diameter of 0.11 ± 0.087 mm, which were dispersed in the haemolymph (Fig. 9 A), or adhered to tissues or organelle walls (Figs. 9 B–F). Most of these granulomas presented yeast aggregates with a high degree of melanization and tissue necrosis (Figs. 9 E and F).

The larvae infected with the VEN C6 isolate presented granulomas with diameters of up to 0.072 ± 0.027 mm in the upper and lower regions. Biofilm-like aggregates of yeast were found adhered to tissue (Fig. 10 A) or inside of organelles (Figs. 10 D and E).

A different infection pattern was observed with *C. albicans,* which showed a greater number of yeast aggregates, tissue invasion and biofilm-like formation. Yeasts and hyphae/pseudohyphae were found invading the tissue (Fig. 11 A) or invading the intestinal wall in the transitional region (Figs. 11 D and E). Additionally, presence of tissue infiltration was observed (Fig. 11 B) together with the biofilm-like formed structures in the intestinal wall and invading other tissue/organs and forming a yeast displacement channel (Fig. 11 D). Granulomas with diameters of up to 0.1 ± 0.038 mm were observed; furthermore, dispersed yeasts or hyphae/pseudohyphae were observed within granulomas with a high degree of melanization (Fig. 11).

## Discussion

In this study, *C. auris* InP13 (clade I) and *C. albicans* were identified as the most virulent strains, exhibiting high melanin production and granuloma formation, which contributed to invasive infection. *C. albicans* showed a pronounced capacity for aggregation, biofilm, and tissue invasion, which may explain the high melanization and mortality observed. Moreover, the InP13 and VEN C6 isolates (classified in the Clade I and Clade IV, respectively) formed pronounced biofilm-like structures in *G. mellonella* tissues, supporting the findings of Hernando-Ortiz et al. [17] who reported that less virulent aggregative strains often developed biofilms. Similarly, Sherry et al. [18] observed increased biofilm formation in aggregative isolates. This underscores the high virulence potential of some *C. auris* strains, which could correlate with pathogenicity. Indeed, the aggregating phenotype has been associated with altered interactions with the immune response, modulation of biofilm production profiles as well as antifungal resistance profile [19].

The virulence characteristics of *C. auris* can be largely attributed to its enzymes arsenal, which includes proteinases and phospholipases, as well as its capability to form biofilm, haemolytic capacity, resistance to environmental stress, and pronounced antifungal resistance [17]. These mechanisms are differentially expressed among the different strains and clades, where independent studies have been confirmed the heterogenicity in enzyme production. As an example, phospholipase activity was detected in 37% of the isolates analysed by Larkin et al. [20] while proteinase secretion was observed in 67% of the isolates depending on the strain and clade analysed. Furthermore, *C. auris* exhibited virulence characteristics associated with its cell wall, such as polymorphism, resistance to abiotic surfaces, adaptation, immune system evasion, and tolerance to external conditions. The cell wall of this species is composed of an outer mannan layer that plays an important role in protecting the internal 1,3-β glucan, which hinders recognition by the host’s immune system and the ability to attack it with antifungal agents, resulting in greater resistance to antifungal agents [21].

The discovery of a new clade (IV) suggests that the genetic population exhibit significant diversity and that a non-recent speciation event or events led to the genesis of each of these separate clades [22]. According to Fayed et al. [23], the diversity of *C. auris* occurs not only between clades but also within the same clade. Whole genome sequencing of different isolates from the same clade (III) showed that they are genetically distinct with a significant high number of single nucleotide polymorphisms. Furthermore, the response to antifungal agents also showed diversity; thus, of the 17 clinical isolates from clades I and III exposed to low and high concentrations of fluconazole, it was possible to observe genomic modifications, including aneuploidy, karyotype alterations and point mutations. Finally, 19 clinical isolates showed genomic differences in genes related to resistance. One of the differences between the clades is related to distinct mutations and/or mechanisms. For example, clades I, III, and IV present point mutations in the *ERG11* gene related to azole resistance, while copy number variations are seen in Clade II [24].

Regarding the virulence analysis, Hernando-Ortiz et al. [17] used a *G. mellonella* model where larvae were inoculated with a yeast suspension of different isolates of *C. auris* (phylogenetically close to the isolates of clade III) ranging from 10^5^ to 10^7^ yeast/larva; they found a dose-dependent mortality, which is consistent with the results of the present study with the higher inoculum (10^6^) reducing larval survival. The comparative study of virulence among *Candida* species, as examined by Garcia-Bustos et al. [16] and using *G. mellonella* as a model, reported to *C. auris* as significantly virulent, resulting in 83% of lethality within three days post-infection. *C. albicans*, however, demonstrated superior pathogenicity, achieving a 95% mortality rate in the same studied period. Notably, no-aggregative *C. auris* strains exhibited greater virulence in comparison with those aggregative strains. In the study of Romera et al. [25] *C. auris* strains from clinical samples caused higher larval mortality and higher melanization scores, the authors tested a *C. albicans* strain, finding it more virulent than *C. auris.* Furthermore, the authors reported that although, no differences in larval mortality were observed between aggregative and non-aggregative *C. auris* strains, the later strains exhibited higher melanization scores compared to aggregative strains.

Melanization has been recognized as important virulence mechanism and was also investigated in this study. Interestingly, no differences in the melanization process were observed among *C. auris* isolates. These results suggest that, while melanization is a relevant virulence factor, its expression may vary significantly across different clades; nonetheless, the non-significant difference in the melanization of infected larvae in this study may be related to the limited number of isolates used per clade (only one isolate). Thus, Garcia-Bustos et al. [16] reported that *C. albicans* achieved 100% melanization in the *G. mellonella* larvae within 24 h of infection, whereas the non-aggregative *C. auris* strains Cj198 and Cj175 showed significantly lower melanization capacity; contrarywise, Romera et al. [25] found that clinical isolates of *C. auris* with aggregative capacity showed increased melanization. It is important to note that melanin could be produced in response to the stress caused by the infection; thus, this compound exhibits an antimicrobial potential and is considered an innate defence mechanism in response to pathogens during the initial infection [26]. The immune response in *G. mellonella* is mediated by prophenoloxidase, which is predominantly found in haemocytes and enocytoids. Once the pathogens are detected, a cascade is activated that results in melanin synthesis [27]. This melanization serves as a mechanism against pathogenic invasion. More recently, Anower et al. observed that three resistant clade I isolates caused high mortality of *G. mellonella* larvae, while a drug-susceptible clade II isolate caused low larval mortality. Finally, melanization was more significant in groups infected with more pathogenic isolates [28].

Additionally*, G. mellonella* larvae display other strategies against infection, such as the recruitment of immune cells to the site of infection and the production of cytokine-like proteins. The interaction of C-type lectins and imulectin-3 on haemocyte membranes with fungal wall antigens facilitates phagocytosis and phagosome formation within haemocytes leading to the destruction of the microbes. Following phagosome formation, the yeast is exposed to reactive oxygen species, acid degradation and enzymatic hydrolysis within phagolysosomes, which effectively neutralize the fungal threat. The inflammatory response, characterized by the formation of granulomas containing melanized or necrotic tissue, further indicates an active immune process [29, 30].

Histopathological examination of *G. mellonella* revealed granulomas with yeast aggregates adhering to organ tissues, the tracheal region and the haemolymph. *Galleria*, in contrast to mammals with a closed circulatory system, has an open one that permeates the entire larval body, especially the abdomen, facilitating the transport of lipids and proteins, the neutralization of endotoxins and the distribution of antimicrobial molecules, essential for larval survival [11, 31]. Both *C. auris* and non-*C. auris* species demonstrated the ability to form melanized granulomas, with a higher prevalence on the extremities. This observation needs further investigation to clarify why a greater number of granulomas were found between the head and tail. Thus, Vasquez-Muñoz et al. [32] observed that *C. auris* aggregates are found dispersed in the tissue of *G. mellonella*, characterizing the pattern of infection and invasion. Bravo-Ruiz et al. [33] suggested that *C. auris* could develop rudimentary pseudohyphae under stressful conditions as a defence mechanism against immune responses. Smith et al. [14] documented melanin-encapsulated *C. auris* pseudohyphae in the tracheal region of *G. mellonella* and the presence of melanin in the haemolymph adjacent to invasive pseudohyphae. In our study, conventional light microscopy revealed enlarged cells approximately 8 μm in diameter. The significant presence of granulomas was also reported in a recent study. Overall, over a 24-h period, 5 to 10 granulomas per profile were observed in a group of larvae infected with *C. auris* 16-1 and more than 50 granulomas per profile in groups infected with *C. auris* 17-1 and 18-1 [28].

Therefore, the results indicate a considerable difference in the levels of pathogenicity and virulence among the *C. auris* clades, which is an important factor for further investigation and the development of alternative methods with therapeutic potential that can more effectively encompass all clades of the species. Furthermore, the results may contribute to a better understanding of the pathogenesis of *C. auris* and its behaviour in response to a host, even if it is an alternative invertebrate *in vivo* model.

## Conclusions

Our study indicates that *C. auris* presents heterogeneous virulence among isolates from different clades or regions. This variation may be related to the aggregative phenotype or clinical origin, as isolates from clade I (InP13) and clade IV (VEN C6) demonstrate higher virulence and a greater ability to form biofilm-like structures in *G. mellonella* compared to clade II (JAP 1) and clade III (SP96).

Additionally, our findings highlight differences in the infection mechanisms of *C. auris* and *C. albicans*. While *C. auris* strains form a greater number of agglomerated yeasts and are involved in haemocytes, *C. albicans* and the InP13 isolate (clade I) show greater invasiveness, with the presence of filaments or pseudohyphae (*C. albicans*).

## References

[1] Ahmad S, Alfouzan W. *Candida auris*: epidemiology, diagnosis, pathogenesis, antifungal susceptibility, and infection control measures to combat the spread of infections in healthcare facilities. Microorganisms. 2021. 10.3390/microorganisms9040807. 33920482 10.3390/microorganisms9040807 PMC8069182

[2] Marena GD, dos Ramos MA, S, Carvalho GC, et al. Development and characterization of an amphotericin B - loaded nanoemulsion applied to *Candida auris* biofilms control. J Drug Deliv Sci Technol. 2022;74:103566.

[3] Centre for Disease Prevention E. Survey on the epidemiological situation, laboratory capacity and preparedness for *Candidozyma (Candida) auris*, 2024.

[4] Marena GD, Carvalho GC, Monazzi LCS, et al. Infection caused by *Candida auris*: state of the art. Mycosphere. 2022;13(1):820–61.

[5] Suphavilai C, Ko KKK, Lim KM, et al. Detection and characterisation of a sixth *Candida auris* clade in Singapore: a genomic and phenotypic study. Lancet Microbe. 2024;5(9). 10.1016/S2666-5247(24)00101-0 39008997

[6] Center of Disease Control and Prevention (CDC) (2018) Infection Prevention and Control for Candida auris|Candida auris|Fungal Diseases|CDC

[7] Jeffery-Smith A, Taori SK, Schelenz S, et al. *Candida auris*: a review of the literature. American society for microbiology; 2018. 10.1128/CMR.00029-17 PMC5740969 29142078

[8] Sanyaolu A, Okorie C, Marinkovic A, et al. *Candida auris*: an overview of the emerging drug-resistant fungal infection. Korean society of infectious diseases, korean society for antimicrobial therapy, Korean society for AIDS; 2022. 10.3947/ic.2022.0008 PMC9259907 35794716

[9] Ruiz-Gaitán AC, Cantón E, Fernández-Rivero ME, Ramírez P, Pemán J. Outbreak of *Candida auris* in Spain: a comparison of antifungal activity by three methods with published data. Int J Antimicrob Agents. 2019;53(5):541–6. 30769198 10.1016/j.ijantimicag.2019.02.005

[10] Tsai CJY, Loh JMS, Proft T. *Galleria mellonella* infection models for the study of bacterial diseases and for antimicrobial drug testing. Virulence. 2016;7(3):214–29. 26730990 10.1080/21505594.2015.1135289 PMC4871635

[11] Wojda I, Staniec B, Sułek M, Kordaczuk J. The greater wax moth *Galleria mellonella*: biology and use in immune studies. Pathog Dis. 2020;78(9):1–15. 10.1093/femspd/ftaa057 PMC7683414 32970818

[12] Li DD, Deng L, Hu GH, et al. Using *Galleria mellonella-Candida albicans* infection model to evaluate antifungal agents. Biol Pharm Bull. 2013;36(9):1482–7. 23995660 10.1248/bpb.b13-00270

[13] Junqueira JC, Mylonakis E, Borghi E. *Galleria mellonella* experimental model: advances and future directions. Pathog Dis. 2021;79(5):1–2. 10.1093/femspd/ftab021 33889960

[14] Smith DFQ, Dragotakes Q, Kulkarni M, Hardwick JM, Casadevall A. *Galleria mellonella* immune melanization is fungicidal during infection. Commun Biol. 2022;5(1):1364. 36510005 10.1038/s42003-022-04340-6 PMC9744840

[15] Mesa-Arango AC, Forastiero A, Bernal-Martínez L, Cuenca-Estrella M, Mellado E, Zaragoza O. The non-mammalian host *Galleria mellonella* can be used to study the virulence of the fungal pathogen *Candida tropicalis* and the efficacy of antifungal drugs during infection by this pathogenic yeast. Med Mycol. 2013;51(5):461–72. 23170962 10.3109/13693786.2012.737031

[16] Garcia-bustos V, Ruiz-saurí A, Ruiz-gaitán A, Sigona-giangreco IA. Characterization of the differential pathogenicity of *Candida auris* in a *Galleria mellonella* infection model. Microbiol Spectr. 2021;9:1–13. 10.1128/spectrum.00013-21 PMC8552516 34106570

[17] Hernando-Ortiz A, Mateo E, Perez-Rodriguez A, de Groot PWJ, Quindós G, Eraso E. Virulence of *Candida auris* from different clinical origins in *Caenorhabditis elegans* and *Galleria mellonella* host models. Virulence. 2021;12(1):1063–75. 33843456 10.1080/21505594.2021.1908765 PMC8043173

[18] Sherry L, Ramage G, Kean R, Borman A, Johnson EM, Richardson MD. Biofilm-forming capability of highly virulent, multidrug-resistant *Candida auris*. Emerg Infect Dis. 2017;23(2):328–31. 28098553 10.3201/eid2302.161320 PMC5324806

[19] Alvarruiz J, Ruiz-Gaitán AC, Cabanero-Navalon MD, et al. Phenotypic impact and multivariable assessment of antifungal susceptibility in *Candida auris* survival using a *Galleria mellonella* model. J Fungi. 2025;11(6):406. 10.3390/jof11060406 PMC12193718 40558919

[20] Larkin E, Hager C, Chandra J, Mukherjee PK. The emerging pathogen *Candida auris*: growth phenotype, virulence factors, activity of antifungals, and effect of SCY-078, a novel glucan synthesis inhibitor, on growth morphology and biofilm formation. Antimicrob Agents Chemother. 2017;61:e02396-16. 28223375 10.1128/AAC.02396-16 PMC5404565

[21] Dakalbab S, Hamdy R, Holigová P, et al. Uniqueness of *Candida auris* cell wall in morphogenesis, virulence, resistance, and immune evasion. Elsevier GmbH; 2024. 10.1016/j.micres.2024.127797 38851008

[22] Gifford H, Rhodes J, Farrer RA. The diverse genomes of *Candida auris*. Elsevier Ltd; 2024. 10.1016/S2666-5247(24)00135-6 38889739

[23] Fayed B, Lazreg IK, AlHumaidi RB, et al. Intra-clade heterogeneity in *Candida auris*: risk of management. Springer; 2023. 10.1007/s00284-023-03416-8 37486431

[24] Smithgall MC, Kilic A, Weidmann M, et al. Genetic and phenotypic intra-clade variation in *Candida auris* isolated from critically ill patients in a New York city tertiary care center. Clin Chem. 2025;71(1):185–91. 39749502 10.1093/clinchem/hvae185

[25] Romera D, Aguilera-Correa JJ, Garciá-Coca M, et al. The *Galleria mellonella* infection model as a system to investigate the virulence of *Candida auris* strains. Pathog Dis. 2020;78(9):1–7. 10.1093/femspd/ftaa067 33098293

[26] Kashem SW, Kaplan DH. Skin immunity to *Candida albicans*. Elsevier Ltd; 2016. 10.1016/j.it.2016.04.007 PMC4931795 27178391

[27] Pereira TC, de Barros PP, de Oliveira Fugisaki LR, et al. Recent advances in the use of *Galleria mellonella* model to study immune responses against human pathogens. J Fungi. 2018. 10.3390/jof4040128. 10.3390/jof4040128 PMC6308929 30486393

[28] Anower MdR, Dennis E, Chaturvedi S, Chaturvedi V. *Candida auris* isolates from New York outbreak are highly pathogenic with measurable experimental disease in *Galleria mellonella*. Microbiol Spectr. 2025. 10.1128/spectrum.02942-23. 39912700 10.1128/spectrum.02942-23 PMC11878041

[29] Smith DFQ, Casadevall A. Fungal immunity and pathogenesis in mammals versus the invertebrate model organism *Galleria mellonella*. Pathog Dis. 2021;79(3):1–25. 10.1093/femspd/ftab013 PMC7981337 33544836

[30] Wrońska AK, Kaczmarek A, Sobich J, Boguś MI. The effect of infection with the entomopathogenic fungus *Conidiobolus coronatus* (Entomopthorales) on eighteen cytokine-like proteins in *Galleria mellonella* (Lepidoptera) larvae. Front Immunol. 2024. 10.3389/fimmu.2024.1385863. 38774871 10.3389/fimmu.2024.1385863 PMC11106378

[31] Mak P, Zdybicka-Barabas A, Cytryńska M. A different repertoire of *Galleria mellonella* antimicrobial peptides in larvae challenged with bacteria and fungi. Dev Comp Immunol. 2010;34(10):1129–36. 20558200 10.1016/j.dci.2010.06.005

[32] Vazquez-Munoz R, Lopez FD, Lopez-Ribot JL. Bismuth nanoantibiotics display anticandidal activity and disrupt the biofilm and cell morphology of the emergent pathogenic yeast *Candida auris*. Antibiotics Basel. 2020;9(8):1–15. 10.3390/antibiotics9080461 PMC7460268 32751405

[33] Bravo Ruiz G, Ross ZK, Gow NAR, Lorenz A. Pseudohyphal growth of the emerging pathogen *Candida auris* is triggered by genotoxic stress through the S phase checkpoint. mSphere. 2020. 10.1128/mSphere.00151-20. 32161147 10.1128/mSphere.00151-20 PMC7067593

